# Human genetics of leishmania infections

**DOI:** 10.1007/s00439-020-02130-w

**Published:** 2020-02-13

**Authors:** Jenefer M. Blackwell, Michaela Fakiola, Léa C. Castellucci

**Affiliations:** 1 Telethon Kids Institute, The University of Western Australia, Nedlands, WA Australia; 2 Department of Pathology, University of Cambridge, Cambridge, UK; 3 INGM-National Institute of Molecular Genetics “Romeo Ed Enrica Invernizzi” Milan, Milan, Italy; 4 National Institute of Science and Technology in Tropical Diseases, Salvador, Brazil; 5 Federal University of Bahia, Salvador, Brazil

## Abstract

Identifying genetic risk factors for parasitic infections such as the leishmaniases could provide important leads for improved therapies and vaccines. Until recently most genetic studies of human leishmaniasis were underpowered and/or not replicated. Here, we focus on recent genome-wide association studies of visceral leishmaniasis (VL) and cutaneous leishmaniasis (CL). For VL, analysis across 2287 cases and 2692 controls from three cohorts identified a single major peak of genome-wide significance (*P*_combined_ = 2.76 × 10^–17^) at *HLA-DRB1*–*HLA-DQA1*. HLA-DRB1*1501 and DRB1*1404/DRB1*1301 were the most significant protective versus risk alleles, respectively, with specific residues at amino acid positions 11 and 13 unique to protective alleles. Epitope-binding studies showed higher frequency of basic AAs in DRB1*1404-/*1301-specific epitopes compared to hydrophobic and polar AAs in DRB1*1501-specific epitopes at anchor residues P4 and P6 which interact with residues at DRB1 positions 11 and 13. For CL, genome-wide significance was not achieved in combined analysis of 2066 cases and 2046 controls across 2 cohorts. Rather, multiple top hits at *P* < 5 × 10^–5^ were observed, amongst which *IFNG-AS1* was of specific interest as a non-coding anti-sense RNA known to influence responses to pathogens by increasing IFN-γ secretion. Association at *LAMP3* encoding dendritic cell lysosomal associated membrane protein 3 was also interesting. LAMP3 increases markedly upon activation of dendritic cells, localizing to the MHC Class II compartment immediately prior to translocation of Class II to the cell surface. Together these GWAS results provide firm confirmation for the importance of antigen presentation and the regulation of IFNγ in determining the outcome of *Leishmania* infections.

**Electronic supplementary material:**

The online version of this article (10.1007/s00439-020-02130-w) contains supplementary material, which is available to authorized users.

## Introduction

The leishmaniases are a group of related diseases caused by parasites of the genus *Leishmania* (order Kinetoplastidae, family Trypansomatidae). The major clinical presentations of leishmaniasis are visceral leishmaniasis (VL) caused by members of the *L. donovani* species complex originating in the Old World, and various forms of cutaneous leishmaniasis (CL) caused by a wide variety of Old and New World species. All species of *Leishmania* are transmitted by sand flies, *Phlebotomus *spp. in the Old World and *Lutzomyia longipalpis* in the New World. Humans, wild animals and domestic animals are known to act as reservoir hosts. VL is characterized by fever, weight loss, epistaxis, pancytopenia, anorexia, abdominal pain, cough, diarrhoea, nausea and vomiting, weakness, fatigue, splenomegaly, hepatomegaly and lymphadenopathy. As well as being a severe systemic infection, a proportion of patients treated for VL go on to present with cutaneous disease termed post-Kala-Azar dermal leishmaniaia (PKDL) (Zijlstra and el-Hassan 2001a; Zijlstra et al. 1994). The World Health Organization estimates that 200,000–400,000 new cases occur annually, 90% of which occur in India, Bangladesh, Sudan, South Sudan, Ethiopia and Brazil (Alvar et al. 2012). The most common form of CL is characterized by localized skin lesions, mainly ulcers, on exposed parts of the body. While normally self-limiting, the degree of pathology and the rate of healing varies, and the lesions leave life-long scars. Mucosal leishmaniasis (ML) and disseminated leishmaniasis (DL) are generally preceded by localized CL, the same species of Leishmania causing both types of disease. ML leads to partial or total destruction of mucous membranes of the nose, mouth and throat. Over 90% of ML is associated with *L. braziliensis* infection in Bolivia, Brazil, Peru and Paraguay in the New World, and with *L. aethiopica* in Ethiopia in the Old World.

Only a small percentage of individuals infected with *Leishmania* parasites go on to develop clinical disease. This variability in rates of clinical disease is determined by a complex interplay between parasite, host and vector genetic factors, as well as environmental and socioeconomic risk factors (Badaro et al. 1986; Chakravarty et al. 2019; Hasker et al. 2018; Jeronimo et al. 1994; Peacock et al. 2001; Reithinger et al. 2007; Shaw et al. 1995; Zijlstra et al. 1994). Immunologically we know a *Leishmania*-specific cellular immune response is a correlate of infection control in resistant individuals, measured traditionally as a positive delayed type hypersensitivity skin test responses in vivo (Follador et al. 2002; Gomes-Silva et al. 2007; Jeronimo et al. 1994; Llanos Cuentas et al. 1984) or lymphocyte proliferation responses ex vivo (Castes et al. 1989; Ho et al. 1982). However, the different forms of CL are generally associated with exaggerated cellular immune responses as measured by these parameters, and for both CL and VL we know that a fine balance exists between the pro-inflammatory cytokines tumour necrosis factor (TNF) and interferon-γ (IFNγ) and anti-inflammatory interleukin-10 (IL-10) (Faria et al. 2005a; Gautam et al. 2011; Gomes-Silva et al. 2007; Kumar et al. 2014; Nylen et al. 2007; Nylen and Sacks 2007; Oliveira et al. 2014; Singh et al. 2012; Zijlstra and el-Hassan 2001b). Thus, it is the balance between antigen-specific pro-inflammatory and anti-inflammatory cytokine responses that are important in determining the outcome of infection with *Leishmania* parasites. A major interest has been in determining the extent to which host genetic factors determine these responses.

One problem in identifying genetic risk factors for complex parasitic infections [reviewed (Abel et al. 2014; Blackwell 2010; Blackwell et al. 2009; Burgner et al. 2006; Loeb 2013)] is that most studies undertaken to date have been underpowered. This makes it difficult to evaluate chance statistical events from real genetic associations. In previous reviews (Blackwell 2010; Burgner et al. 2006; Castellucci et al. 2014; Mohamed et al. 2019) we provided summary tables and/or comprehensive supplementary web tables that listed all the studies undertaken in humans up to that time looking for susceptibility genes for protozoan parasite infections, including leishmaniasis. It is not our intention to repeat that exercise here. Rather, we focus on how recent genome-wide approaches using well-powered sample sizes have uncovered a divergence in the way they highlight specific genes and pathways involved in susceptibility to the two major forms of visceral versus cutaneous disease.

### Visceral leishmaniasis

**Fig. 1:**
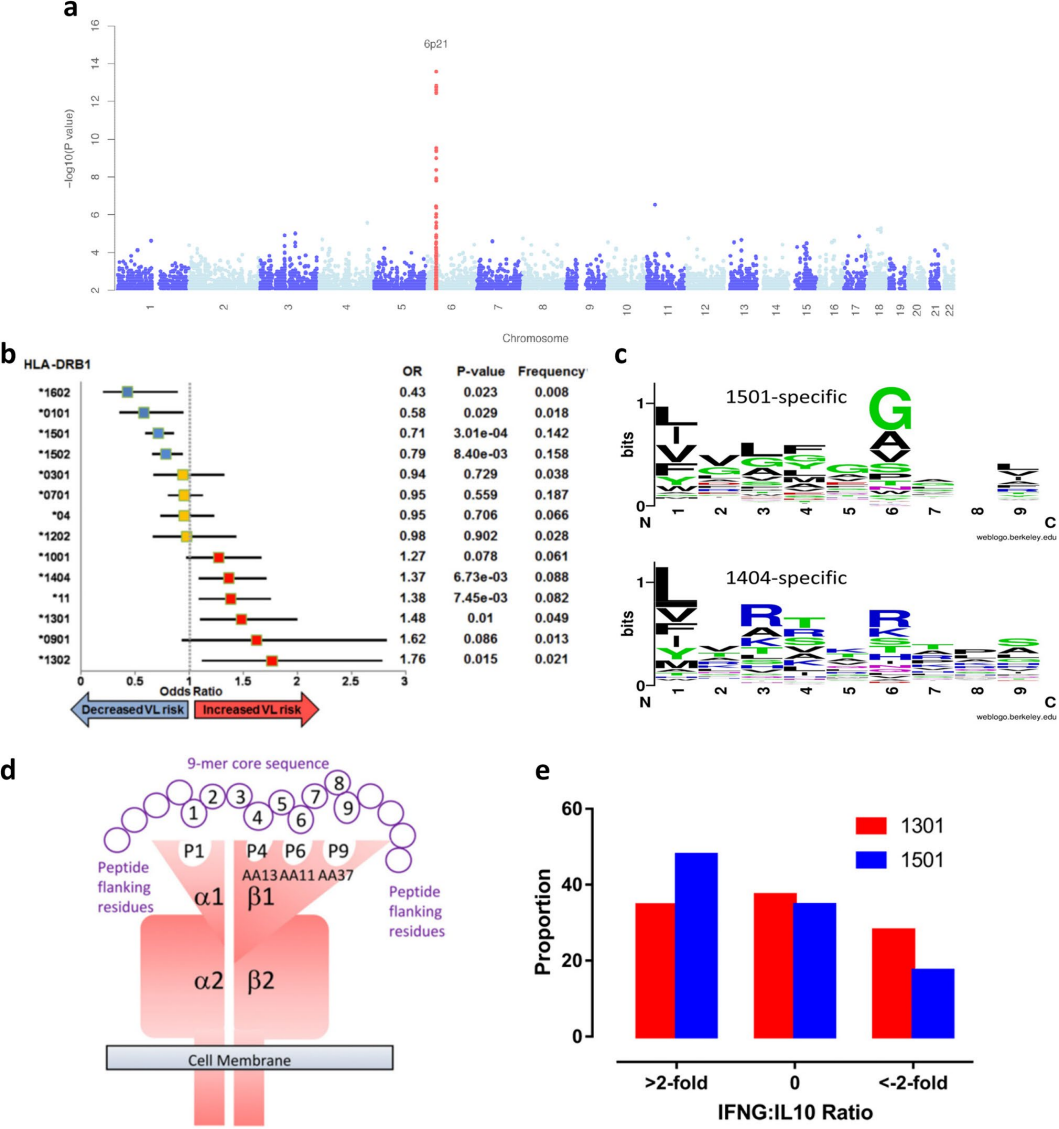
Journey from GWAS to function in understanding the role of HLA-DRB1 as the single important genetic risk factor in VL. **a** Manhattan plot for meta-analysis across independent cohorts of VL caused by *L. donovani* in India and *L. infantum/chagasi* in Brazil (Fakiola et al. 2013). **b** Forest plot showing associations between VL and classical DRB1 alleles (Singh et al. 2018). **c** WebLogo plots for sequence motifs that characterize 9-mer cores for epitopes binding preferentially to the protective DRB1*1501 compared to the DRB1*1401 risk allele (Singh et al. 2018). Polar amino acids are green (G, S, T, Y, C) or purple (Q, N), basic amino acids are blue (K, R, H), acidic amino acids are red (D, E), and hydrophobic amino acids are black (A, V, L, I, P, W, F, M). The size of the letter indicates the frequency with which the amino acid is found in this position of core 9-mers, and the overall peak height on the *y*-axis indicates the degree of conservation for specific epitopes at this location. Greater promiscuity and a higher frequency of basic amino acids in DRB1*1401-specific epitopes, compared with hydrophobic and polar amino acids in DRB1*1501-specific epitopes, at anchor residues P4 and P6 which interact with residues at DRB1 positions 11 and 13. **d** Model of epitope binding to the DRA/DRB alpha/beta dimer demonstrating the specific interaction between amino acids at positions 4 and 6 in the 9-mer cores of *Leishmania* antigenic epitopes that bind to pockets 4 and 6 created by amino acids at positions 11 and 13 in the DRB1 molecule. **e** Shows the higher ratio of IFNG:IL10 in cytokine responses to peptides based on epitopes captured dendritic cells homozygous for the protective DRB1*1501 allele compared to epitopes captured from dendritic cells homozygous for the DRB1*1301 risk allele (Singh et al. 2018)

Complex segregation analysis of familial visceral leishmaniasis (VL) caused by *L. chagasi* in Brazil favored all genetic models over a sporadic model for disease susceptibility, with support for all single locus models over polygenic or multifactorial models of susceptibility (Peacock et al. 2001). While a single locus model might seem unlikely given the complex nature of a parasitic infectious disease, our recent GWAS of VL (Fakiola et al. 2013) (Fig. 1a), conducted as part of phase 2 of the Wellcome Trust Case–Control Consortium, highlighted the polymorphic HLA-DR-DQ region within the major histocompatibility complex of immune-related genes as the single major genetic determinant of VL (*P*_combined_ = 2.76 × 10^17^; odds ratio = 1.41; 95% confidence interval = 1.30–1.52 over three cohorts). This replication across three independent cohorts crossed the geographical and epidemiological divides of continent and etiological parasite species, *L. donovani* in India and *L. infantum/chagasi* in Brazil. This robust demonstration that the most important genetic risk factor for VL lies at the heart of eliciting host CD4 + T-cell-mediated immunity against *Leishmania* parasites has major implications for vaccine design, allowing researchers to pursue single-minded post-GWAS functional analyses designed to understand the host–parasite interaction at a high resolution molecular level of antigen presenting cell function. Indeed, fine mapping (Fig. 1b) demonstrated that HLA-DRB1*1501 and DRB1*1404/DRB1*1301 were the most significant protective versus risk alleles, respectively, with specific residues at amino acid positions 11 and 13 unique to protective alleles (Singh et al. 2018). To determine the nature of epitopes binding to risk versus protective DRB1 alleles both in silico and in vitro experimental approaches were employed. NetMHCIIPan2.1 (Nielsen et al. 2010) was used to map epitopes and binding affinities across 49 *Leishmania* vaccine candidates, and across peptide epitopes captured from dendritic cells treated with crude *Leishmania* antigen and identified using mass spectrometry and alignment to a reference *Leishmania* genome (Singh et al. 2018). In these studies, greater peptide promiscuity was observed in sequence motifs for 9-mer core epitopes predicted to bind to risk (*1404/*1301) compared to protective (*1501) DRB1 alleles. In addition, there was a higher frequency of basic AAs in DRB1*1404-/*1301-specific epitopes compared to hydrophobic and polar AAs in DRB1*1501-specific epitopes (Fig. 1c) at anchor residues P4 and P6 which interact with residues at DRB1 position 11 and 13 (Fig. 1d). Peptides prepared based on sequences of epitopes captured from risk versus protective alleles were then used in whole blood cytokine assays to determine whether these differences in epitope-binding affected the important balance between pro- and anti-inflammatory cytokines. Cured VL patients made variable but robust IFN-γ, TNF and IL-10 responses to 20-mer peptides based on captured epitopes, with peptides based on DRB1*1501-captured epitopes resulting in a higher proportion (odds ratio 2.23; 95%CI 1.17–4.25; *P* = 0.017) of patients with IFN-γ:IL10 ratios > twofold compared to peptides based on DRB1*1301-captured epitopes (Fig. 1e). These genetic studies have, therefore, led to a greater understanding of the molecular interactions between host antigen presenting cell molecules and antigenic epitopes of the pathogen that drive the immune response towards either a protective or a disease-associated response.

GWAS data for the Indian and Brazilian cohorts were also examined separately using a less stringent cutoff of *P* < 1 × 10^–5^ to look for potential population-specific associations. None of the regions associated at *P* < 1 × 10^–5^ in these separate analyses showed significant association in the combined analysis. Of the hits that were population-specific (one for India; 13 for Brazil), 8 were in intergenic regions and the remainder landed in genes with no obvious functional significance (Table S1).

### Cutaneous leishmaniasis

Human family-based genetic epidemiology studies of CL disease caused by *L. peruviana* were consistent with a gene by environment multifactorial model, with a two-locus model of inheritance providing the best fit to the data (Shaw et al. 1995). This suggested that major genetic risk factors might also readily be found for CL disease. A GWAS was considered the best way to test this hypothesis. Genome-wide genetic analysis was undertaken (Castellucci et al. 2020) using DNAs from 2066 cases of CL caused by *L. braziliensis* and 2046 controls across two independent cohorts. The rarer forms of ML and DL disease caused by *L. braziliensis* were not used in this study due to lack of statistical power. Linear mixed models were employed in FastLMM (Lippert et al. 2011) to take account of relatedness, multiple testing and population heterogeneity. Combined analysis across these cohorts that were similarly powered to the VL study found no associations that achieved genome-wide significance, commonly accepted as *P* < 5 × 10^–8^ for studies employing variants with minor allele frequencies > 0.05 (Dudbridge and Gusnanto 2008; Fadista et al. 2016; Peer et al. 2008). Rather, multiple top hits at *P* < 5 × 10^–5^, consistent with complex disease inheritance, were observed that included some plausible novel candidate susceptibility genes. Amongst these were *SERPINB10* (*P*_combined_ = 2.67 × 10^–6^), *CRLF3* (*P*_combined_ = 5.12 × 10^–6^), *STX7* (*P*_combined_ = 6.06 × 10^–6^), *KRT80* (*P*_combined_ = 6.58 × 10^–6^), *LAMP3* (*P*_combined_ = 6.54 × 10^–6^) and *IFNG-AS1* (*P*_combined_ = 1.32 × 10^–5^). Effect sizes for these associations were small (odds ratios 1.07–1.21) (Castellucci et al. 2020) and lower than those observed for the top single nucleotides HLA variant associated with VL (odds ratio 1.49–1.50 Indian discovery; 1.38–1.67 Brazilian discovery) (Fakiola et al. 2013). To obtain functional support for these associations, published expression data (Novais et al. 2015) available on GEObase (GSE55664) was interrogated. *LAMP3*, *STX7* and *CRLF3* were all shown to be expressed more highly in CL biopsies compared to normal skin, whereas expression of *KRT80* was strongly reduced (Castellucci et al. 2020). *SERPINB10* was not expressed in skin, while probes were not available on the chip employed (Novais et al. 2015) for *IFNG-AS1*. Nevertheless, *IFNG-AS1* was of specific interest as a non-coding anti-sense RNA known to influence responses to pathogens by increasing IFN-γ secretion in T cells and NK cells (Collier et al. 2012; Gomez et al. 2013; Petermann et al. 2019). Association at *LAMP3* encoding dendritic cell lysosomal associated membrane protein 3 was also interesting. LAMP3 increases markedly upon activation of dendritic cells, localizing to the MHC Class II compartment immediately prior to translocation of Class II to the cell surface (de Saint-Vis et al. 1998). Together these GWAS results strengthen our knowledge of the importance of antigen presentation and the regulation of IFNγ in determining the outcome of *Leishmania* infections.

### Do GWAS data provide support for previous candidate gene studies?

Data from the VL and CL GWAS provide the opportunity to evaluate earlier candidate gene studies which, as suggested above, were frequently compromised by lack of statistical power and/or were not replicated. Prior to the GWAS, family-based genome-wide linkage studies together with studies in mouse models had highlighted putative non-HLA candidate susceptibility genes for VL. These included *CXCR2*, *CXCR1*, *SLC11A1*, *DLL1*, *CCL1*, *CCL16*, and *IL2RB* (Bucheton et al. 2007; Fakiola et al. 2011; Jamieson et al. 2007; Mehrotra et al. 2011; Mohamed et al. 2004). None of these loci showed consistent association in the Indian or Brazilian cohorts used for the GWAS analysis, even when looking at precisely the same single nucleotide variant (Fakiola et al. 2013). Four of the original candidate gene studies were undertaken in The Sudan, which could indicate some geographic or ethnic heterogeneity in genetic risk factors for VL and/or differences in the parasite. On balance, however, it seems unlikely that variants in these genes remain strong candidates as genetic risk factors for VL.

For CL, candidate gene studies (Almeida et al. 2015; Cabrera et al. 1995; Castellucci et al. 2006, 2010, 2011, 2012, 2014; Ramasawmy et al. 2010; Salhi et al. 2008) undertaken in *L. braziliensis* endemic regions had demonstrated associations with polymorphisms at multiple genes associated with pro- and anti-inflammatory responses (*TNFA*, *SLC11A1*, *CXCR1*, *IL6*, *IL10, CCL2/MCP1*) and/or with wound healing (*FLI1*, *CTGF*, *TGFBR2*, *SMAD2*, *SMAD3*, *SMAD7*, *COL1A1*) in determining susceptibility to CL or ML disease. This included susceptibility genes identified from murine studies of leishmaniasis (*SLC11A1*, *FLI1*) (Castellucci et al. 2010, 2011). Although frequently underpinned by functional data (Castellucci et al. 2006; Ramasawmy et al. 2010; Salhi et al. 2008) and/or supported by prior immunological studies (Bacellar et al. 2002; Castes et al. 1993; D'Oliveira et al. 2002; Faria et al. 2005b; Lessa et al. 2001), these studies had also generally lacked statistical power. Given a priori evidence to look at these genes in the GWAS data, a value of *P*_combined_ < 0.01 was used as a cutoff to identify possible associations. No variants were associated at *P*_combined_ < 0.01 for *TNFA*, *SLC11A1*, *CXCR1*, *IL6*, *IL10*, *CCL2/MCP1*, *FLI1*, *CTGF*, *COL1A1*, or *TGFBR2* in the CL GWAS data (Castellucci et al. 2020). This failure to replicate may, in some cases, be due to the fact that only the CL phenotype was examined in the GWAS, and not ML or DL disease. Associations were observed for variants at *SMAD2* (*P* = 1.47 × 10^–4^), *SMAD3* (*P* = 0.009) and *SMAD7* (*P* = 0.005), wound healing genes that had previously been shown to be associated with CL (Castellucci et al. 2012). Some evidence for associations at related SMADs was also observed, specifically at *SMAD1* (*P* = 7.49 × 10^–4^), *SMAD4* (*P* = 1.90 × 10^–4^), *SMAD6* (*P* = 0.001), and *SMAD9* (*P* = 0.004). The major role for SMAD proteins is to transduce signals from receptors of the transforming growth factor beta superfamily. Although no association at *P*_combined_ < 0.01 was observed at *TGFBR2*, association at the functionally related gene *TGFBR3* (*P* = 3.98 × 10^–4^) was supported at multiple variants across the gene. Similarly, associations observed at collagen genes, *COL24A1* (*P* = 2.06 × 10^–4^) and *COL11A1* (*P* = 6.22 × 10^–4^), functionally related to wound healing gene *COL1A1*, were supported at multiple variants across each gene. Associations were also observed for variants at genes, *IL6R* (*P* = 7.14 × 10^–4^) and *IL10R* (*P* = 0.003), encoding receptors for cytokines IL-6 and IL-10 that have previously been shown to be genetically or functionally associated with CL. On balance, results of the CL GWAS provided more support for previously identified candidate genetic risk factors than had the VL GWAS data.

## Conclusions

The results of these GWAS studies provide contrasting stories in relation to genetic risk factors for VL compared to CL disease. Whereas a single association at genome-wide significance was observed for VL (Fakiola et al. 2013), the results of the CL GWAS showed multiple potential genetic risk factors all with small effect sizes and none achieving genome-wide significance (Castellucci et al. 2020). The latter is consistent with polygenic inheritance for a multifactorial complex infectious disease. Much larger studies would be required to determine whether any of these putative susceptibility loci would ever achieve genome-wide significance. To better understand the pathogenesis of VL and CL disease, it is likely that a more cost-effective approach will be to use the current GWAS data in combination with functional expression studies that can be carried out on fewer individuals. This will certainly be true for the rarer forms of disease such as ML, DL or PKDL, where obtaining the sample size required to achieve genome-wide significance in genetic studies would be very difficult. This does not, however, preclude the possibility that future large-scale sequencing projects may become affordable and reveal yet uncovered genetic risk factors, especially in the context of these rarer forms of disease.

## Electronic supplementary material

Below is the link to the electronic supplementary material.

Supplementary file1 (PDF 204 kb)
