# Supplementary material for: Human genetics of leishmania infections
Source: Hum Genet. 2020 Feb 13;139(6):813–9. doi: 10.1007/s00439-020-02130-w (PMC7272388; doi:10.1007/s00439-020-02130-w)
Supplement: Supplementary file 1 — Supplementary file1 (PDF 204 kb) [file 439_2020_2130_MOESM1_ESM.pdf]

## Supplementary Table 1 (2 pages)

| | | | Allele | | Brazilian discovery | | | Indian discovery | | | Consequence | Gene Symbol | Gene Name |
|---|---|---|---|---|---|---|---|---|---|---|---|---|---|
| chr | rsID | position | 0 | 1 | P value | Beta | SE | P value | Beta | SE | | | |
| **(A) India-specific** | | | | | | | | | | | | | |
| 2 | rs2312548 | 69809264 | A | C | 0.407 | -0.08 | 0.09 | 2.24E-06 | 0.30 | 0.06 | intronic | ANXA4 | Annexin IV |
| | | | | | | | | | | | | | |
| **(B) Brazil-specific** | | | | | | | | | | | | | |
| 2 | rs3748934 | 100376340 | A | G | 4.88E-06 | -19.56 | 4.32 | 0.014 | 2.71 | 1.10 | synonymous | CHST10 | Carbohydrate sulfotransferase 10 |
| 4 | rs1355967 | 10836785 | A | C | 1.40E-07 | -0.46 | 0.09 | 0.169 | 0.09 | 0.06 | intergenic | CLNK/HS3ST1 | Cytokine dependent hematopoietic cell linker/Heparan sulfate-glucosamine 3-sulfotransferase 1 |
| 4 | rs902174 | 185892165 | A | G | 4.60E-06 | -0.42 | 0.09 | 0.902 | 0.01 | 0.06 | missense | CENPV | Centromere protein V |
| 7 | rs2527214 | 158447342 | A | G | 5.98E-06 | -0.60 | 0.13 | 0.743 | -0.03 | 0.09 | intergenic | WDR80/VIPR2 | Autophagy related 16 like 2/Vasoactive intestinal peptide receptor 2 |
| 10 | rs2674355 | 126052643 | A | G | 4.01E-06 | 0.85 | 0.19 | 0.060 | -0.46 | 0.24 | intergenic | CHST15/OAT | Carbohydrate sulfotransferase 15/Ornithine aminotransferase |
| 11 | rs2957710 | 10334795 | A | G | 3.18E-06 | 0.49 | 0.11 | 0.918 | 0.01 | 0.06 | intronic | CAND1.11 | Uncharacterized gene |
| 11 | rs1484433 | 15756036 | A | C | 7.62E-06 | -0.41 | 0.09 | 0.262 | -0.08 | 0.07 | intergenic | INSC/SOX6 | INSC spindle orientation adaptor protein/SRY-box transcription factor 6 |
| 11 | rs11031947 | 32691220 | A | G | 3.83E-06 | 0.41 | 0.09 | 0.684 | -0.03 | 0.07 | intronic | CCDC73 | Coiled-coil domain containing 73 |
| 11 | rs4078355 | 124436544 | A | G | 3.39E-06 | 0.52 | 0.11 | 0.948 | 0.00 | 0.07 | intergenic | CCDC15/SLC37A2 | Coiled-coil domain containing 15/Solute carrier family 37 member 2 |
| 15 | rs1549520 | 48256580 | A | C | 3.06E-06 | -0.45 | 0.10 | 0.123 | 0.10 | 0.06 | intronic | ATP8B4 | ATPase phospholipid transporting 8B4 |

## Supplementary Table 1 (2 pages)

| | | | | | | | | | | | | | |
|---|---|---|---|---|---|---|---|---|---|---|---|---|---|
| 15 | rs8036138 | 83693160 | A | G | 8.26E-06 | 0.41 | 0.09 | - | - | - | intergenic | PDE8A/AKAD13 | Phosphodiesterase 8A/A-kinase anchoring protein 13 |
| 18 | rs8098585 | 36762038 | A | G | 6.37E-07 | 0.46 | 0.09 | 0.575 | 0.04 | 0.07 | intergenic | CELF4/PIK3C3 | CUGBP Elav-like family member 4/Phosphati-dylinositol 3-kinase catalytic subunit type 3 |
| 18 | rs12454166 | 53780638 | A | G | 3.96E-06 | 0.60 | 0.13 | 0.775 | -0.03 | 0.10 | intergenic | NARS1/NEDD4L | Asparaginyl-tRNA synthetase 1/NEDD4 like E3 ubiquitin protein ligase |

**Table S1.** GWAS data for the Indian and Brazilian cohorts used in the VL GWAS (Fakiola et al. 2013) examined separately using a less stringent cut-off of $P<1x10^{-5}$ to look for potential population-specific associations. None of the regions associated at $P<1x10^{-5}$ in these separate analyses showed significant association in the combined analysis. Of the hits that were population-specific (1 for India shown in part A; 13 for Brazil shown in part B), 8 were in intergenic regions and the remainder landed in genes with no obvious functional significance. The Betas and allele frequency are shown for Allele 1. Positions are in NCBI human genome Build 36. Where the consequence of the variant is intergenic, the nearest protein-coding genes on either side are shown. Of the genes listed CLNK (also known as MIST) has possible significance for its role in the regulation of immunoreceptor signalling, including PLC-gamma-mediated B cell antigen receptor signalling and FC-epsilon R1-mediated mast cell degranulation (Taken from NCBI: (Cao et al., 1999 [PubMed 10562326]; Goitsuka et al., 2000, 2001 [PubMed 10744659] [PubMed 11463797])). However, the associated variant rs1355967 lies some 540kb downstream of CLNK, where it lies 167kb upstream of HS3ST1.

Fakiola M, Strange A, Cordell HJ, Miller EN, Pirinen M, Su Z, Mishra A, Mehrotra S, Monteiro GR, Band G, Bellenguez C, Dronov S, Edkins S, Freeman C, Giannoulatou E, Gray E, Hunt SE, Lacerda HG, Langford C, Pearson R, Pontes NN, Rai M, Singh SP, Smith L, Sousa O, Vukcevic D, Bramon E, Brown MA, Casas JP, Corvin A, Duncanson A, Jankowski J, Markus HS, Mathew CG, Palmer CN, Plomin R, Rautanen A, Sawcer SJ, Trembath RC, Viswanathan AC, Wood NW, Wilson ME, Deloukas P, Peltonen L, Christiansen F, Witt C, Jeronimo SM, Sundar S, Spencer CC, Blackwell JM, Donnelly P (2013) Common variants in the HLA-DRB1-HLA-DQA1 HLA class II region are associated with susceptibility to visceral leishmaniasis. Nat Genet 45: 208-13. doi: ng.2518 [pii] 10.1038/ng.2518
